# Loss of EHF facilitates the development of treatment-induced neuroendocrine prostate cancer

**DOI:** 10.1038/s41419-020-03326-8

**Published:** 2021-01-05

**Authors:** Zhi Long, Liang Deng, Chao Li, Qiangrong He, Yao He, Xiheng Hu, Yi Cai, Yu Gan

**Affiliations:** 1 Department of Urology, Andrology Center, The Third Xiangya Hospital, Central South University, Changsha, Hunan 410013 People’s Republic of China; 2 Department of Urology, Xiangya Hospital, Central South University, Changsha, Hunan 410008 People’s Republic of China

**Keywords:** Transdifferentiation, Prostate cancer

## Abstract

The rising of a highly aggressive subtype of castration-resistant prostate cancer (CRPC) named treatment-induced neuroendocrine prostate cancer (t-NEPC) after androgen deprivation therapy (ADT) is well known for its features of the neuroendocrine differentiation (NED) and androgen receptor (AR) independence. However, t-NEPC is still largely unknown. Here, we found that EHF is notably depressed in t-NEPC tumors, patient-derived xenografts, transgenic mice, and cell models. Results from cell lines uncovered that ADT represses EHF expression, which is required for the ADT-induced NED. Mechanism dissection revealed that ADT decreases the EHF transcription via relieving the AR binding to different androgen-responsive elements, which then promotes the expression and enzymatic activity of enhancer of zeste homolog 2 (EZH2), consequently catalyzing tri-methylation lysine 27 of histone H3 for transcriptional repression of its downstream genes to promote the NED. Furthermore, preclinical studies from cell and mice models proved that recovery of EHF expression or using EZH2 inhibitor can attenuate aggressive properties of CRPC cells, hinder the progression of t-NEPC, and promote the response of CPRC cells to enzalutamide. Together, we elucidate that the ADT/AR/EHF/EZH2 signaling is required for the ADT-enhanced NED and plays a critical role in the progression of t-NEPC.

## Introduction

Prostate cancer (PCa) is the most prevalent cancer and the second leading cause of cancer death in men in the United States^1^. As the progression of PCa is generally driven by androgen, systematic androgen deprivation therapy (ADT) becomes the gold standard for treating advanced PCa^2,3^. However, although the majority of PCa patients initially have a positive response to ADT, resistance develops inevitably leading to castration-resistant PCa (CRPC), accompanied by a poor prognosis^2–4^. Most tumors in CRPC stage still rely on androgen receptor (AR) signaling through AR amplification, mutation, alternative splicing, or other means^2,3^. Next-generation ADT drugs such as enzalutamide (ENZ) and abiraterone thus have been introduced in the treatment of CPRC tumors for their potent AR antagonism^5,6^. Although these agents extend survival, the response is temporary as further resistance to their use eventually causes disease progression^2–7^.

In a subset of CRPC patients (up to 15–20%), therapeutic resistance is associated with the emergence of neuroendocrine (NE) PCa (NEPC)^7–9^. In this setting, under the selective pressure from highly potent AR-targeted therapies, PCa cells gradually reduce the dependency on AR signaling, lose their original luminal epithelial identity, and acquire a NE phenotype^2–4,7^. This phenotype reprogramming occurs through lineage plasticity, a biological process mediated, in part, by the pluripotency transcriptional factor SOX2, and facilitates cellular proliferation, metastasis, and drug resistance^10–14^. Histologically, treatment-related NEPC (t-NEPC) tumors present as pure small cell morphology or diversity morphologies with both small cells and adenocarcinoma cells mixed^8,9,15^. t-NEPC tumors universally express NE makers such as enolase 2 (ENO2), chromogranin A (CHGA), and synaptophysin (SYP), and exhibit an AR-independent state characterized by reduced or none AR expression^8,9,15^. Therefore, patients developing t-NEPC react indolently to AR-targeted therapies and have to be treated with platinum-based cytotoxic agents. The prognosis is far from satisfactory, with a median overall survival of 8.9 months (pure small cell carcinoma) and 26.1 months (mixed histology) from t-NEPC diagnosis^9^. This emphasizes the necessity for searching druggable therapy targets for this lethal disease. Nevertheless, the options available are limited for the molecular basis underlying the formation of t-NEPC from prostate adenocarcinoma (AdPC) remains unclear.

EHF belongs to the epithelial-specific ETS (ESE) transcriptional factor family that plays a critical role in the pathogenesis of PCa^16–19^. EHF encodes a 300-amino acid protein named ESE-3, with a highly endogenous expression in normal prostate tissue to maintain cell homeostasis of prostate epithelial cells and restrict them in a well-differentiated condition^16,18^. Loss of EHF expression induces epithelial–mesenchymal transition (EMT) and cell dedifferentiation, and confers to prostate epithelial cells a stem-like phenotype, along with aggressive and tumor-initiating properties^16,18^. Furthermore, knockdown of EHF in PCa cells promotes cell migration and survival, and contributes to taxol resistance^16–18,20^. Loss of EHF is also linked to elevated expression of pluripotency markers and indicates a poor prognosis in PCa tumors^16–18,21,22^. However, the regulatory mechanism underpinning of downregulation of EHF expression in PCa is still largely unexplored.

Since EHF plays a pivotal role in restraining prostate epithelial cells or PCa cells in a luminal epithelial phenotype, whether EHF loss contributes to the progression of CRPC by conferring PCa cells lineage plasticity and inducing phenotype reprogramming has aroused our interest. In this study, we analyzed the expression profile of EHF in t-NEPC tumors and preclinical models, including patient-derived xenografts (PDXs), genetically engineered mouse models (GEMMs), and cell models. We also used in vitro and in vivo models to demonstrate the critical role of EHF loss and the underlying molecular mechanism in t-NEPC development.

## Results

### EHF expression is downregulated in t-NEPC

**Fig. 1:**
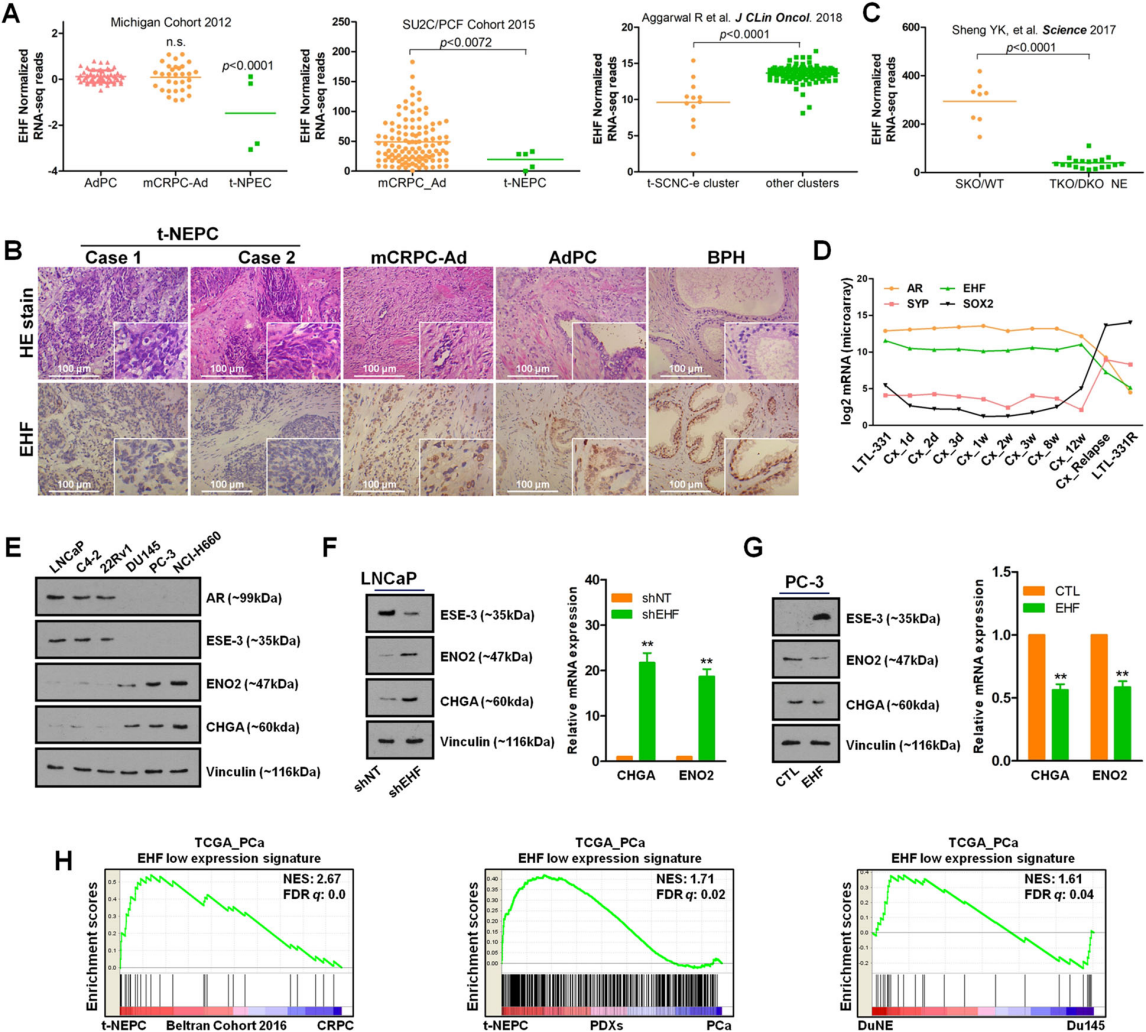
EHF expression is downregulated in t-NEPC. **A** EHF mRNA expression of tumor tissues derived from Michigan 2012^25^, SU2C/PCF 2015^26^, and Aggarwal et al.^8^ clinical prostate cancer cohorts was plotted. **B** EHF expression in tumor or prostate tissues from t-NEPC, CRPC-Ad, AdPC, and BPH patients was measured by immunohistochemistry. Scale bar 100 μm. **C** EHF mRNA expression from the GEMM models^11^ was plotted. **D** EHF, AR, SYP, and SOX2 mRNA expressions during the progression of AdPC (LTL-331) to t-NEPC (LTL-331R) by castration surgery to the host mice^23^ were plotted. **E** AR, EHF, ENO2, and CHGA protein expressions in LNCaP, C4-2, 22Rv1, DU145, PC-3, and NCI-H660 cell lines were measured by immunoblotting. **F** ENO2 and CHGA levels in LNCaP cells with/without EHF knockdown were measured by qPCR and immunoblotting. **G** ENO2 and CHGA levels in PC-3 cells with/without EHF overexpression were measured by qPCR and immunoblotting. **H** GSEA revealed the enrichment of the “EHF low expression-specific genes” signature into the transcriptome of t-NEPC tumors^7^, t-NEPC PDXs^23^, and NEPC DuNE cell line^12^ in comparison with their prostate adenocarcinoma counterparts. All qPCR and immunoblotting assays were repeated in triplicate. The two-tailed Student’s *t* test or one-way ANOVA, followed by Dunnett’s *t* test was used to compare results between two groups with ** denoting *p* < 0.01. Bar graphs show means ± SD. AdPC localized prostate adenocarcinoma tumors, mCRPC metastatic castration-resistant prostate cancer, Ad adenocarcinoma, t-NEPC treatment-induced neuroendocrine prostate cancer, t-SCNC-e treatment-induced small cell neuroendocrine cancer-enriched, PCa prostate cancer, BPH benign prostate hyperplasia, TCGA The Cancer Genome Atlas, HE hematein–eosin, SKO/WT wild-type and single *Pten* loss, TKO/DKO *Pten* plus *Trp53* double loss and *Pten*, *Trp53* plus *Rb1* loss, NE neuroendocrine, PDXs patient-derived xenografts, n.s. not significant.

The emergence of t-NEPC has become a major clinical concern as this subtype of CRPC tumors is highly aggressive and represents a poor survival^8,9^. To profile the expression of EHF in t-NEPC, we firstly analyzed the publicly available RNA-sequencing (RNA-Seq) or microarray datasets from patient tumors, PDXs, GEMMs, and cell models^8,11,23–26^. We found that EHF expression was significantly lower in t-NEPC tumors than in metastatic CRPC (mCRPC-Adenocarcinoma) and localized AdPC tumors (Michigan 2012 dataset)^25^; a similar result was also validated in the SU2C/PCF mCRPC dataset (Fig. 1A, left and middle panels)^26^. In another study of unsupervised hierarchical clustering of the transcriptional profile of mCRPC biopsy specimens, a specific t-NEPC-enriched cluster was identified^8^, and we further confirmed that EHF expression was remarkably lower in this cluster than in other mCRPC clusters (Fig. 1A, right panels). We also confirmed the loss of EHF in t-NEPC tumor tissues (Fig. 1B). As expected, the positive association of EHF expression with prostate-specific antigen (a classic AdPC maker) and the negative association of EHF with ENO2 and SOX2 (t-NEPC markers) were observed in mCRPC tumors (Fig. S1a).

Consistently, RNA-Seq data from the GEMMs under ADT showed that the tumors from both wild-type (WT) and *Pten* loss (SKO) mice expressed higher EHF, while the tumors from *Pten*/*Trp53* (DKO) and *Pten*/*Trp53*/*Rb1* knockout (TKO) mice harbored NE features^11^ and expressed extremely lower EHF (Fig. 1C). There was also a positive correlation of EHF loss with t-NEPC formation in PDXs modeling the evolution from AdPC to t-NEPC^23^. Specifically, the pre-castrated LTL-331 PDX initially exhibited a classic adenocarcinoma phenotype and gradually transformed to the t-NEPC LTL-331R PDX after surgical castration, during the process EHF was notably depressed and positively associated with AR, and negatively with SYP and SOX2 (Fig. 1D).

We then tested EHF expression in a panel of five PCa cell lines and found that EHF was highly expressed in the LNCaP, C4-2, and 22Rv1 AdPC cell lines, but relatively depressed in the androgen-insensitive DU145 cells as well as the PC-3^27^ and NCI-H660 NEPC cell lines (Fig. 1E), which was consistent with the microarray data from these cancer cells reported by Barretina et al. ^24^ (Fig. S1b). We also knocked down EHF in the LNCaP cells (Fig. 1F) and introduced EHF into the PC-3 cells (Fig. 1G), and further confirmed that EHF negatively regulated the expression of ENO2 and CHGA at both the mRNA and protein level. Interestingly, when a proved NEPC driver gene *SRRM4*^12^ was exogenously introduced into PCa cells, tumor cells almost without EHF expression, just like DU145 cells, could acquire a pluripotency gene network that could not be induced by *SRRM4* in cells with EHF endogenous expression (Fig. S1b). This highlights the possible role of EHF in restricting lineage plasticity.

Finally, we employed the TCGA (The Cancer Genome Atlas) PCa RNA-Seq dataset^26^ and defined an EHF low expression-specific gene signature based on a stratified analysis according to the expression level of EHF (Table S1). Gene set enrichment analysis (GSEA) showed that this gene signature was strongly enriched into the transcriptome of t-NEPC tumors^7^, t-NEPC PDXs^23^, and NEPC DuNE cell line^12^ (Fig. 1H). Moreover, GSEA analysis of the TCGA data (Table S2) further revealed that PCa tumors with low EHF expression had similar characteristics with t-NEPC in alleviating the dependency on AR signaling and gaining stem cell features (Fig. S1c). Low EHF expression also indicated a poor prognosis (Fig. S1d). Together, these results demonstrated that EHF is downregulated in a subset of t-NEPC, suggesting a functional significance of EHF loss in t-NEPC formation.

### ADT-induced EHF depression is important for NE differentiation

**Fig. 2:**
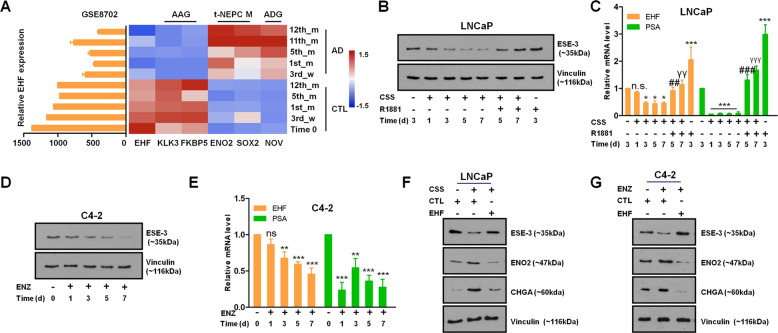
ADT-induced EHF depression is important for NE differentiation. **A** Bar graph showing EHF expression (left) and heatmap of AR-associated genes including EHF, t-NEPC markers, and an androgen-depressed gene in long-term androgen-deprived LNCaP cells (GSE8702). **B**, **C** Effects of androgen deprivation (by culturing in CSS medium) and AR ligand R1881 on EHF expression in LNCaP cells were measured by qPCR and immunoblotting. *P* values for each sample were obtained based on nontreated controls (*) and cells cultured in CSS medium for 5 days (^#^) or for 7 days (^γ^) for EHF and PSA, respectively. **D**, **E** Effects of ENZ on EHF expression in C4-2 cells were determined by qPCR and immunoblotting. *P* values for each sample were obtained based on nontreated controls (*) for EHF and PSA. **F** EHF, ENO2, and CHGA protein levels in LNCaP cells with/without EHF overexpression responsive to androgen deprivation (by culturing in CSS medium for 48 h) were measured by immunoblotting. **G** EHF, ENO2, and CHGA protein levels in C4-2 cells with/without EHF overexpression responsive to ENZ treatment for 48 h were measured by immunoblotting. All qPCR and immunoblotting assays were repeated in triplicate. The two-tailed Student’s *t* test or one-way ANOVA, followed by Dunnett’s *t* test was used to compare results between two groups with * denoting *p* < 0.05, ^**/##/γγ^ denoting *p* < 0.01, and ^***/###/γγγ^ denoting *p* < 0.001. Bar graphs show means ± SD. AAG AR-associated genes, M markers, ADG androgen-depressed gene, AD androgen deprivation, CTL control, CSS charcoal-stripped serum, ENZ enzalutamide, n.s. not significant.

Since castration was the only experimental intervention to trigger EHF depression in the LTL-331 PDX, we assumed that EHF might be an androgen-response gene. To verify the hypothesis, we first analyzed a series of publicly available datasets, wherein the androgen-sensitive LNCaP cells were continuously propagated in an androgen-deprived medium (GSE8702), which exhibited reduced expression of EHF and the AR activated genes (e.g., *KLK3* and *FKBP5)* as well as activated expression of the AR-depressed genes (e.g., *NOV*) and the NE makers (e.g., ENO2 and SOX2) (Fig. 2A). As expected, when LNCaP cells were treated with dihydrotestosterone (DHT, GSE7868), the expression of these genes changed in reverse (Fig. S2a). Moreover, DHT also enhanced EHF expression in VCaP cells (GSE51872; Fig. S2b). To further validate the androgen regulation of EHF, we checked its expression in LNCaP and C4-2 cells subjected to AR blockade and/or androgen. Accordingly, ablation of androgen led to elevated expression of EHF at both the mRNA and protein level, while the increased expression was alleviated by the addition of the synthetic androgen reagent R1881 in LNCaP cells (Fig. 2B, C). In addition, treatment with ENZ (a potent AR antagonist) inhibited EHF expression in AR signaling active CRPC C4-2 cells (Fig. 2D, E). We then artificially overexpressed EHF in the LNCaP and C4-2 cells, and cultured these cells with the androgen-deprived medium and ENZ, respectively. The results showed that reverted EHF blocked the emergence of NE markers (e.g., ENO2 and CHGA; Fig. 2F, G), indicating that EHF loss is important for ADT-induced NE differentiation. These data suggest that EHF is an androgen-depressed gene, whose loss is crucial for ADT-induced NE features.

### AR directs transcriptional activation of EHF in PCa cells

**Fig. 3:**
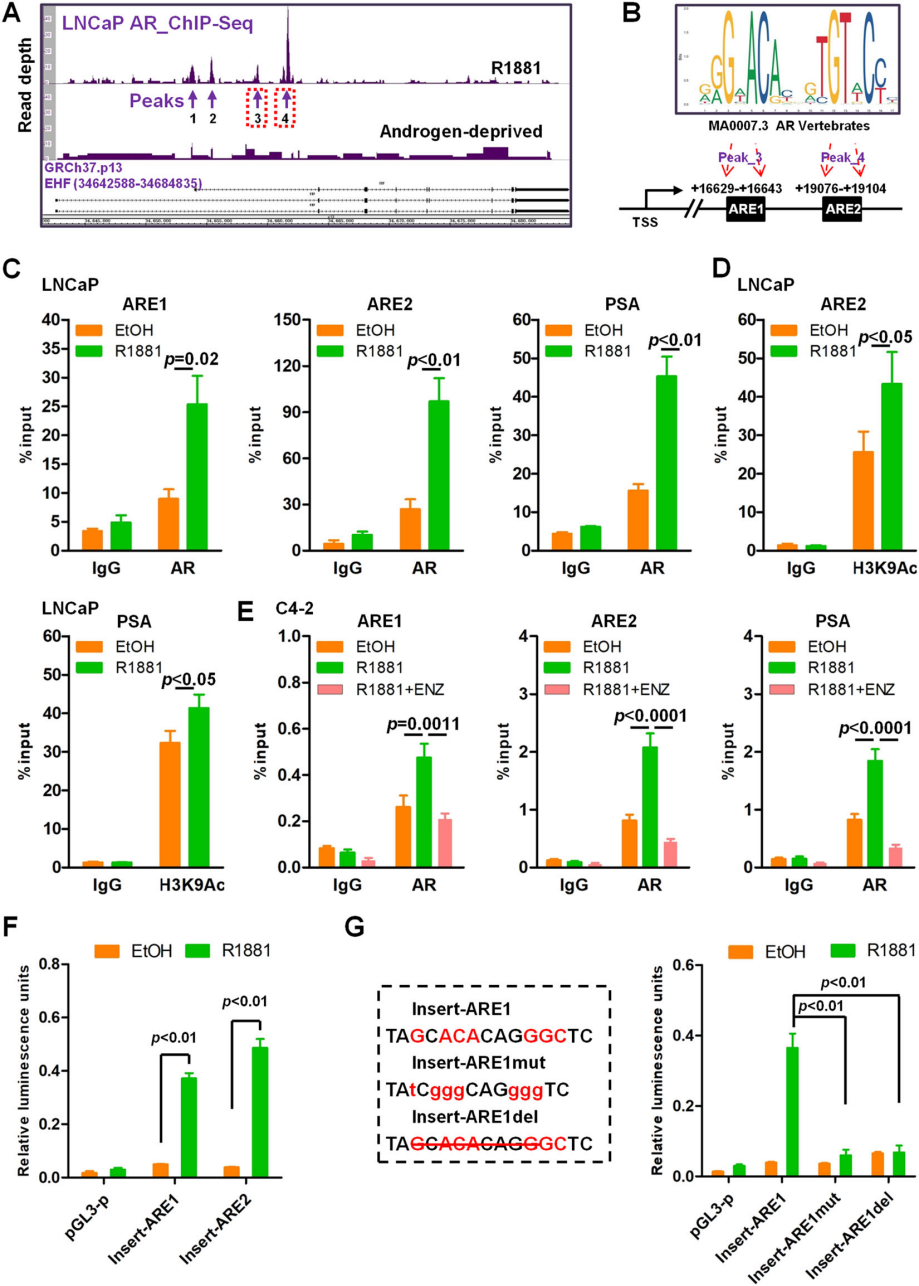
AR directs transcriptional activation of EHF in PCa. **A** ChIP-Seq profiles in androgen-stimulated LNCaP cells (GSE55007) indicating AR enrichment on four genomic sites (peaks 1–4) located in the EHF’s first intron region. **B** Schema showing AR-binding motif obtained from the JASPAR database (top). Bottom panel showing two putative direct AR-binding sites selected from the DNA sequences represented by peaks 1–4. **C** ChIP-qPCR data showing recruitment of AR on the EHF’s first intron upon R1881 (10 nM) stimulation in LNCaP cells. **D** Same condition as in **C**, except H3K9Ac marks on the EHF’s first intron. **E** ChIP-qPCR data indicating enrichment of AR on the EHF’s first intron in R1881 (10 nM)-stimulated C4-2 cells with/without ENZ treatment (10 μM). PSA (KLK3) promoter was employed as a positive control for R1881 or ENZ treatment (**C**, **D**). **F** Transcriptional activation ability of ARE1 and ARE2 as indicated by increased luciferase reporter activity. Luminescence units were normalized by Renilla luciferase signal. **G** Transcriptional inhibitory function of ARE mutants (sequences are presented) was indicated by dual-luciferase reporter assay. Nucleotides in red are sequences of consensus ARE1, mutated ARE1, and deleted ARE1, respectively. The two-tailed Student’s t test or one-way ANOVA, followed by Dunnett’s *t* test was used to compare results between two groups. Experiments were performed with three biologically independent samples. Bar graphs show means ± SD. ARE androgen-responsive element, H3K9Ac H3 lysine 9 acetylation.

The role of AR has been extensively characterized as a transcriptional factor to mediate the effect of androgen. We found that AR could positively regulate the expression of EHF at both the mRNA and protein level; these results agreed with the fact that AR was almost co-expressed with EHF in a synchronous manner in PCa cells (Fig. 1E) and was positively correlated with EHF in several PCa patient cohorts (Fig. S3a). To examine whether EHF is transcriptionally regulated by AR, we analyzed chromatin immunoprecipitation-sequencing (ChIP-Seq) data of AR (GSE55007) and found that AR was recruited to four distinct genomic sites located in the EHF’s first intron region (peaks 1–4; Fig. 3A). We further employed publicly available transcription factor binding prediction software, JASPAR (http://www.jaspar.genereg.net), to screen the DNA sequences represented by these sites. Two putative direct AR-binding sites were selected, which are androgen-responsive element 1 (ARE1) and ARE2 (Fig. 3B).

ChIP-quantitative polymerase chain reaction (ChIP-qPCR) assays for AR in R1881-stimulated LNCaP cells were used to confirm the occupancy of AR on the two predicted AREs, and a marked enrichment for AR-binding at ARE1 and ARE2 was observed (Fig. 3C). Epigenetic modifications around specific genomic loci affect the transcriptional activity of a gene^28^. We found a significant increase in the enrichment of H3K9Ac (H3 lysine 9 acetylation) marks at ARE2 by ChIP-qPCR in the same context (Fig. 3D), indicating the transcriptional activation of EHF in androgen-stimulated LNCaP cells. We also examined the occupancy of AR on ARE1 and ARE2 in ENZ-treated C4-2 cells, and a profound decrease in the recruitment was observed, suggesting impaired AR binding under an androgen deprivation condition (Fig. 3E).

To further confirm the impact of AR binding at the two AREs on transcriptional activity of EHF, we then performed dual-luciferase reporter assays in androgen-sensitive LNCaP cells, wherein two reporter plasmids containing ARE1 and ARE2 were constructed, respectively. The relative luciferase signals from both reporter plasmids were remarkably increased by treatment with R1881 (Fig. 3F). We also constructed reporter plasmids with ARE1 mutated or deleted. These modifications impeded the increase of the luciferase signal responsive to R1881 (Fig. 3G). Together, these findings indicated that AR acts as a direct transcriptional activator of EHF gene, and ADT can attenuate EHF transcriptional activation leading to its downregulation.

### EZH2 is essential for NE phenotype induced by EHF loss

**Fig. 4:**
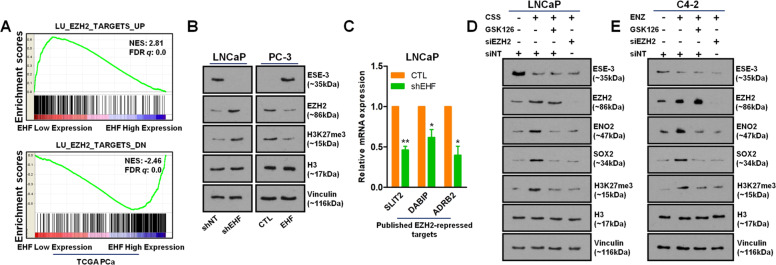
EZH2 is essential for NE phenotype induced by EHF loss. **A** GSEA analysis indicated that EZH2 might function as a downstream effector molecule of EHF in prostate cancer. **B** EHF, EZH2, H3K27me3, and H3 protein expressions in LNCaP cells with/without EHF knockdown and PC-3 cells with/without EHF overexpression were measured by immunoblotting. **C** The mRNA level of three known EZH2-repressed targets (SLIT2, DABIP, and ADRB2) was measured by qPCR in LNCaP cells with/without EHF knockdown. **D** EHF, EZH2, ENO2, SOX2, H3K27me3, and H3 protein levels were measured by immunoblotting in CSS cultured LNCaP cells with/without GSK126 treatment (5 μM) or EZH2 knockdown. **E** Same tests as in **D**, except that measured in ENZ-treated (10 μM) C4-2 cells with/without GSK126 treatment or EZH2 knockdown. The two-tailed Student’s *t* test was used to compare results between two groups with * denoting *p* < 0.05 and ** denoting *p* < 0.01. All qPCR and immunoblotting assays were repeated in triplicate. Bar graphs show means ± SD. DN down, H3K27me3 tri-methylation lysine 27 of histone H3, H3 histone H3.

To explore the signaling networks of EHF loss, we stratified the transcriptome of the TCGA PCa dataset based on EHF expression level and did an unbiased GSEA analysis using the “C2_curated gene sets” collection from the latest MSigDB database (Table S3, 4). The results revealed that the genes upregulated when the enhancer of zeste homolog 2 (EZH2) was knocked down were significantly enriched into the transcriptome of the PCa tumors with low EHF expression, while the genes downregulated were enriched into the high EHF subgroup (Fig. 4A). EZH2 is a major enzymatic component of polycomb repressive complex 2 (PRC2) to catalyze tri-methylation lysine 27 of histone H3 (H3K27me3) for transcriptional repression of the downstream genes. Therefore, we assumed that EZH2 might act as a downstream molecule of EHF. To confirm this, we performed immunoblotting assays and found that EHF negatively controlled the expression and the methyltransferase activity of EZH2 in PCa cells (Fig. 4B). We also performed qPCR analysis for several published EZH2-repressed targets (i.e., SLIT2, DABIP, and ADRB2)^29^ and, as expected, their expressions were significantly depressed when EHF was silenced (Fig. 4C). Moreover, the negative association of EZH2 expression with EHF was observed in mCPRC tumors and PDXs (Fig. S4).

It has been reported that EZH2 expression and its PRC2 activity are tightly implicated in the progression of t-NEPC. We also observed that both the decrease of EHF and increase of EZH2 have coincidently occurred with a NE phenotype in PCa cells subjected to ADT (Fig. 4D, E). However, when the activity of EZH2 was re-abolished by a specific small interfering RNA pool or an EZH2 inhibitor, the NE features were diminished again regardless of EHF loss (Fig. 4D, E). Taken together, EZH2 acts as a downstream target of EHF and is required for NE differentiation induce by EHF loss.

### EHF knockdown promotes the aggressive properties of PCa cells

**Fig. 5:**
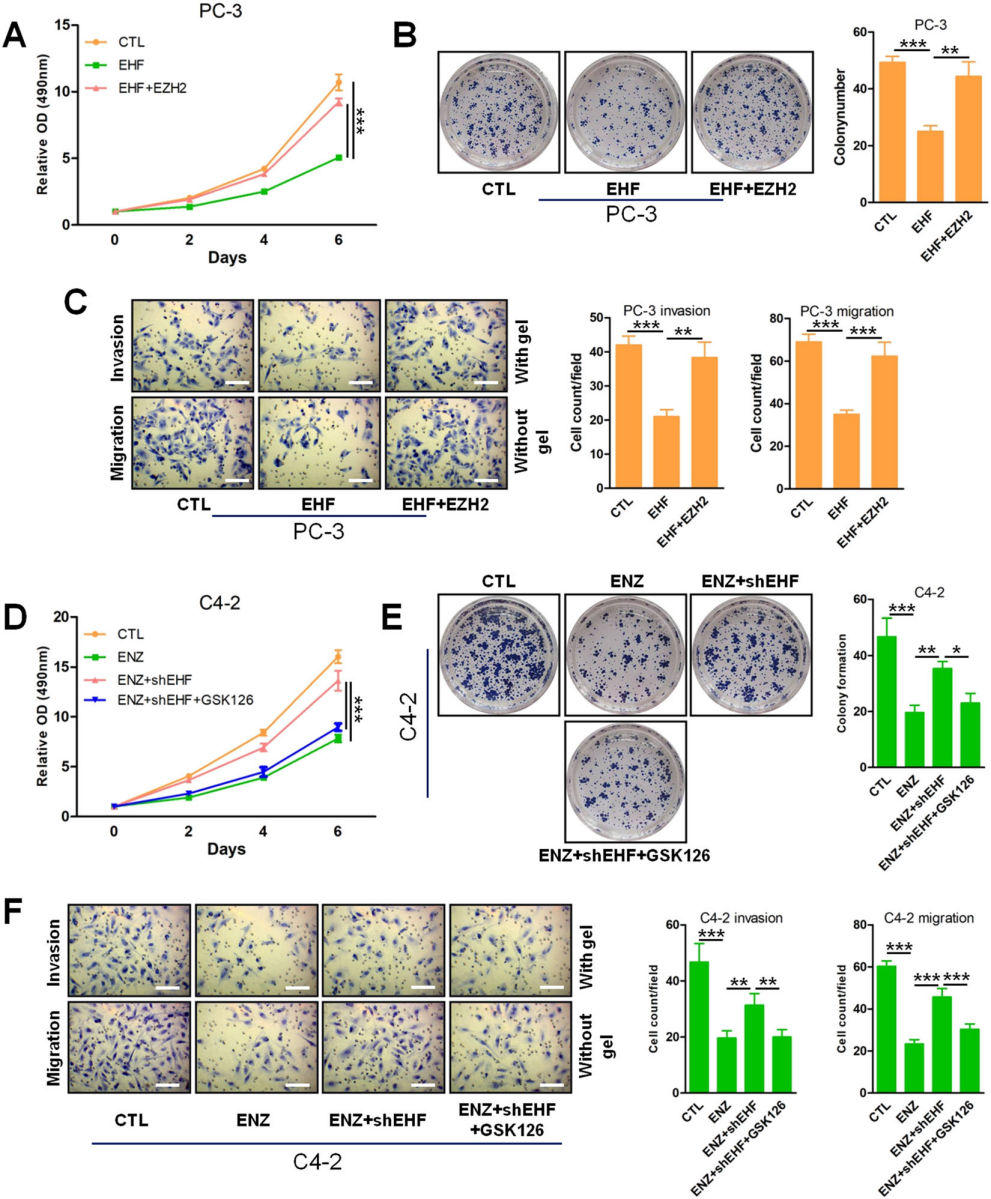
EHF knockdown promotes the aggressive properties of PCa cells. **A** MTS assays measured cell proliferation of PC-3 cells with/without EHF overexpression and PC-3 cells with EHF and EZH2 overexpression. **B** Same cells as in (**A**) were used to perform colony formation assays. Representative images are shown. **C** Same cells as in (**A**) were used to perform cell invasion and migration assays in transwell chambers with/without coating with Matrigel. Representative images are shown. Scale bar 100 μm. **D** C4-2 cells with/without EHF knockdown were treated with/without ENZ (10 μM) and GSK126 (5 μM), and cell proliferation was determined by MTS assays. **E** Colony formation assays were performed in the same context as in **D**. Representative images are shown. **F** Cell invasion and migration assays were performed in the same context as in **D**. Representative images are shown. Scale bar 100 μm. One-way ANOVA, followed by Dunnett’s *t* test was used to compare results between two groups with * denoting *p* < 0.05, ** denoting *p* < 0.01 and ^***^ denoting *p* < 0.001. Experiments were performed with three biologically independent samples. Bar graphs show means ± SD. OD optical density.

Consistent with the reports from other investigators^16^, we also confirmed that EHF functioned as a tumor suppressor gene in CRPC 22Rv1 cell line in which EHF expressed in a moderate level (Fig. 1D and Figs. S5–7). To further define the impact of EHF on NEPC cells, we overexpressed it in PC-3 cells, a PCa cell line with characteristics of NEPC and negative EHF expression^27^. Stable expression of EHF led to profound inhibition of PC-3 cell proliferation, invasiveness, migration, and colony formation (Fig. 5A–C). However, the coexistence of EZH2 and EHF reverted the aggressive features of PC-3 cells in the same context (Fig. 5A–C), further confirming that EZH2 functioned as an effecter of EHF loss. Furthermore, to determine the function of EHF loss to drive ADT resistance, we subsequently silenced EHF in C4-2 cells with EHF endogenous expression. Notably, knockdown of EHF regardless of ENZ led to promoting the growth rate and the invasive properties of C4-2 cells (Fig. 5D–F), suggesting EHF loss contributed to ENZ resistance. As expected, EZH2 inhibitor GSK126 could resensitize C4-2 cells with EHF knockdown to ENZ (Fig. 5D–F). These data together verify that EHF loss is a crucial event of CRPC development, while regaining expression of EHF in NEPC-like cells attenuates their aggressive biological behaviors.

### EHF loss contributes to the progression of CRPC and NE differentiation in mice

**Fig. 6:**
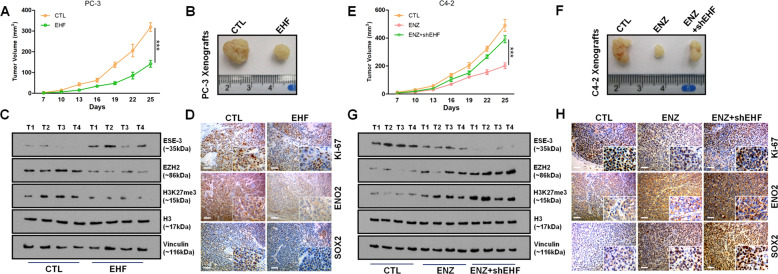
EHF loss contributes to the progression of CRPC and NE differentiation in mice. **A** Growth of tumor xenografts in male nude mice 25 days after subcutaneous inoculation with PC-3 cells with/without EHF overexpression. *n* = 4 mice per group. **B** Representative tumor images in (**A**). **C** EHF, EZH2, H3K27me3, and H3 protein expressions from the xenografts in (**A**) were measured by immunoblotting. **D** Ki-67, ENO2, and SOX2 expressions from the xenografts in (**A**) were measured by immunohistochemistry. Scale bar 100 μm. **E** Growth of tumor xenografts in male nude mice treated with/without ENZ (30 mg/kg, intraperitoneal injection, every other day) 25 days after subcutaneous inoculation with C4-2 cells with/without EHF knockdown. *n* = 4 mice per group. **F** Representative tumor images in (**E**). **G** Same proteins in (**C**) from the xenografts in (**E**) were measured by immunoblotting. (**H**) Same markers in (**D**) from the xenografts in (**E**) were measured by immunohistochemistry. Scale bar 100 μm. The two-tailed Student’s *t* test or one-way ANOVA, followed by Dunnett’s *t* test was used to compare results between two groups with *** denoting *p* < 0.001. Immunoblotting assays were repeated in triplicate. Bar graphs show means ± SD.

To verify all above the in vitro cell lines data in the in vivo mice models, immunodeficient mice bearing PC-3 cells with/without EHF overexpression were employed to assess the impact of EHF on growth and gene expression patterns. Elevated EHF led to a remarkable inhibition of tumor growth (Fig. 6A, B). Moreover, in accord with the in vitro results, gain of EHF caused a decreased expression and an attenuated methyltransferase activity of EZH2 in in vivo models (Fig. 6C). We also evaluated the expression of the proliferation marker Ki-67 and the t-NEPC makers ENO2 and SOX2 by immunohistochemical (IHC) staining in PC-3 tumors. We confirmed that EHF repressed t-NEPC makers while inhibiting the tumor growth (Fig. 6D). Established C4-2 cells with/without EHF loss were also engrafted subcutaneously in mice. Not surprisingly, ENZ induced a decrease of EHF expression (Fig. 6G, top panels). Gene manipulation to further repress EHF allowed C4-2 tumors to resist ENZ (Fig. 6E, F), which was also reflected by the IHC staining of Ki-67 (Fig. 6H, top panels). In addition, ENZ elevated the expression of EZH2 and its enzymatic function (Fig. 6G), while the expression of ENO2 and SOX2 was also enhanced simultaneously in C4-2 xenografts (Fig. 6H, middle and bottom panels). More importantly, EHF inhibition further increased the activity of EZH2 and the expression of t-NEPC markers on the basis of ENZ treatment (Fig. 6G, H). These data collected in in vivo mice models also support the event of EHF loss that plays a critical role in CRPC progression and NE differentiation.

## Discussion

**Fig. 7:**
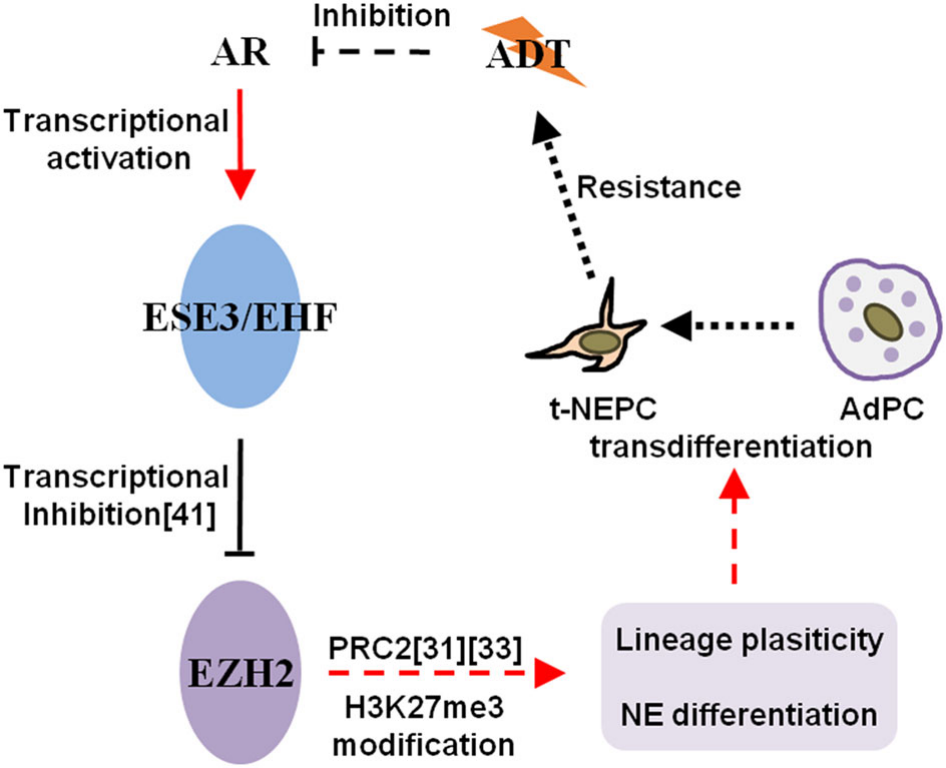
Proposed model of EHF loss-mediated therapeutic resistance and NE differentiation of prostate cancer. AR androgen receptor, ADT androgen deprivation therapy, PRC2 polycomb repressive complex 2, t-NEPC treatment-induced neuroendocrine prostate cancer, AdPC prostate adenocarcinoma, NE neuroendocrine.

EHF restrains normal prostate epithelial cells or PCa cells in a highly differentiated condition with luminal epithelial lineage, while the loss of EHF endows stem-like features to these cells^16–18^. Decreased EHF expression insufficiently explained by methylation of an evolutionarily conserved CPG site in its promoter has been associated with the transformation from prostate epithelial cells to malignant cells^16–19^. This was supported by a previous IHC study, in which a large fraction of high-grade prostate intraepithelial neoplasia (60%) and organ-confined prostate tumors (80%) had a reduced level of EHF when compared to normal prostate^18^. During the tumor-initiating period, cells acquire cancer stem-like cell (CSC) features as a result of elevated expression of key EMT and CSC genes, but the transformed cells still keep a luminal epithelial phenotype^16,17,19,30^. In this study, we further extended the significance of EHF loss to accelerate the progression of t-NEPC and provided an exact mechanism of how EHF was regulated by the androgen level under hormonal therapies (Fig. 7). Based on these findings, we get a better understanding of the distinct ADT resistance mechanism associated with t-NEPC formation.

The transition from AdPC to t-NEPC is finely defined as an adaptive response of PCa cells in order to survive from potent AR-targeted therapies^7,15^. This always happens in a late disease stage, when a subtype of PCa cells regains lineage plasticity by acquiring an EMT and CSC associated gene network to facilitate a NE phenotype switch^10–13^. Here, we provide compelling evidence that EHF loss plays a crucial role during this adaptive process. We prove that AR acts as a transcriptional activator of EHF. ADT inhibits the functional activation of AR and thus leads to the transcriptional repression of EHF. EHF inhibition in return causes ADT resistance in PCa cells by inducing NE differentiation. These results indicate that loss of EHF may occur and play a critical role at least in two distinct stages of PCa disease progression, which are tumor-initiating stage and lineage switch stage. More importantly, the factors driving EHF loss may be different in these two stages. Whether EHF expression could be recovered in the period between the two stages needs to be further explored in a longitudinal PCa progression model.

It is worth noting that some t-NEPC tumors still had endogenous EHF expression (Fig. 1A, left and right panel), this may be explained by the perspective that RNA-Seq results could just represent a snapshot of the whole process of lineage switch. Moreover, the nature of t-NEPC is a heterogeneous tumor disease that arose as a result of multiple context-dependent mechanisms, including genomic aberrations^8,31,32^, epigenetic modifications^31,33^, alternative splicing^34–36^, abnormal expression of transcriptional factors^11–13,36,37^, metabolic shifts^38^, and tumor microenvironment^39^. Therefore, a subset of t-NEPC may be established to bypass the transcriptional repression of EHF and through alternative mechanisms. Interestingly, some CRPC tumors without apparent NE features were observed with a low EHF expression (Fig. 1A, middle panel). This fact suggests that the final shape of t-NEPC tumors needs the cooperation of other factors apart from EHF loss. Consistent with this notion, concomitant *Trp53* and *Rb1* disruption helped to conceal the accessible regions of EHF-DNA-binding motifs in chromatin of an engineered NEPC cell model^40^.

Our results indicate that EZH2 is one of the downstream targets of EHF. This is consistent with a previous report, in which EHF was proved to be a transcriptional repressor of EZH2^41^. We found that EZH2 inhibitor impeded the induction of NE markers and recovered the sensitivity of CRPC cells to ENZ regardless of EHF loss, highlighting the usage of EZH2 inhibitors to revert lineage switch. These results as well as results from other investigators^42^ support the combined use of EZH2 inhibitors and ENZ to treat CPRC and t-NEPC, which are currently under clinical evaluation and promising (NCT02082977, NCT01897571). Efforts to directly revert the expression of EHF may be also beneficial to patients with advanced PCa tumors. Our team recently identified that LIN28B, as one of the four core pluripotency stem cell genes, promotes lineage plasticity and the development of t-NEPC^43^. EHF has been reported to transcriptionally repress LIN28B expression in PCa cells^17^, further highlighting that EHF is a promising therapeutic target for inhibiting lineage plasticity. Furthermore, the activation of interleukin/signal transducer and activator of transcription 3 (IL-6/STAT3) signaling was well implicated in t-NEPC formation^44^. EHF loss-associated IL-6/STAT3 activation also has become possible targets for t-NEPC therapy^21,22^.

In summary, our study reveals a molecular mechanism to explain the formation of t-NEPC, by which ADT attenuates EHF expression, and EHF loss then induces NE phenotype in PCa cells, which finally contributes to ADT resistance.

## Methods

### Patient tissues

The paraffin sections of tumor or prostate tissues from patients used in this study were collected and pathologically diagnosed by the Department of Pathology with approval from the Research Ethics Committee of The Third Xiangya Hospital of Central South University (CSU, 2020-S037) and written informed consent was obtained from each patient.

### RNA-Seq/microarray data

The clinical cohorts used in this study are as follows: RNA-Seq data for the Beltran et al. 2016 cohort (34 mCRPC-Ads, 15 t-NEPCs) was from Weill Medical College of Cornell University^7^; RNA-Seq data for the Michigan 2012 cohort (59 localized PCas, 31 mCRPC-Ads, 4 t-NEPCs), the SU2C/PCF 2015 cohort (113 mCRPC-Ads, 5 t-NEPCs), and the TCGA PCa cohort (493 primary PCas) were accessed through the cBioPortal^25,26,45^; RNA-Seq data for the Aggarwal et al. 2018 cohort was obtained from the supplementary files of the original paper^8^.

Microarray profiles for the LTL-331-331R castration time-series were accessed from the Gene Expression Omnibus database (GSE59986), while the microarray data for GSEA analysis of the transcriptome of PDXs (15 adenocarcinomas, 3 t-NEPCs) was accessed from GSE41193; RNA-Seq data for the GEMMs reported by Ku et al. (WT, SKO, DKO, and TKO) was accessed from GSE90891.

RNA-Seq data for the DuNE cell model was previously reported by the authors of this study^12^. Microarray data for the Cancer Cell Line Encyclopedia was accessed through the Oncomine^24^. Microarray data for analyzing the response of gene expression profile of PCa cell lines (LNCaP, VCaP) to ADT and DHT was accessed from GSE8702, GSE7868, and GSE51872.

### Cell lines and cell culture

All the PCa (LNCaP, C4-2, 22Rv1, Du145, PC-3, and NCI-H660) and human embryonic kidney 293T cell lines were obtained from American Type Culture Collection. Cell culture conditions for these cell lines have been previously described^35^. All cell lines used in the study were authenticated by short tandem repeat profiling and checked negative for *mycoplasma* contamination.

### siRNA and DNA transient transfection

Cells were transfected with ON-TARGETplus SMARTpool (Dharmacon) small interfering RNA (siRNA) for a specific gene or with nontargeting negative control siRNA using Lipofectamine RNAiMAX (Invitrogen). Lipofectamine 3000 (Invitrogen) was used for the introduction of an expression plasmid for a specific gene or empty control vector. Transfection protocols have previously been described^12^. Information of siRNA pools and expression plasmids is described in the Supplementary file.

### Lentivirus production and generation of stable cell lines

The EHF coding sequence was obtained from 293T complementary DNA by PCR and was cloned into the pFUGWBW vector as we previously reported^35^. The pLKO.1 expression vectors used for short hairpin RNA (shRNA) targeting EHF were purchased from Dharmacon, and the best one for efficient gene knockdown was selected. Lentiviruses expression of EHF and its specific shRNA were generated by transfecting the respective plasmids into 293T cells following our previous report^12^. LNCaP, PC-3, and C4-2 cells were infected with lentiviruses encoding control or EHF/shRNA, followed by blasticidin selection to maintain stable cell populations. Information on the Dharmacon vectors is listed in the Supplementary file.

### Real-time qPCR and immunoblotting assays

Real-time qPCR and immunoblotting assays were performed as previously reported^12,46^. Experiments were repeated at least three times. One representative blot was presented for each immunoblotting test. Information on primers and antibodies is listed in the Supplementary file.

### Bioinformatic analysis

As previously reported by our team^12^, GSEA analysis (http://software.broadinstitute.org/gsea/index.jsp) was carried out in the study to determine whether a defined set of genes showed significant and concordant differences between two biological phenotypes (e.g., t-NEPC vs. AdPC) or two groups of tumors (e.g., low EHF expression vs. high EHF expression). The analysis was performed using the latest MSigDB database for each collection or using gene sets curated based on the published data. Phenotype permutation was selected when the sample size was more than five in each group. Otherwise, gene set permutation was applied. Heatmaps in this study were constructed based on the *z*-scores derived from the normalized microarray data.

### Cell proliferation, colony formation, invasion, and migration assays

The cell proliferation assay method was previously described^12^, where cell proliferation rate was evaluated every other day for 6 days post seeding. Colony formation assay was performed as our previous report^12^, where cells were stained after 14 days, and colonies with a size of >1 mm were counted. Cell invasion and migration assays have been previously detailed^47^, where cells penetrating the filter member with/without Matrigel (Corning) were stained and counted. Three independent biological replicates were performed for these assays.

### ChIP-Seq and ChIP

ChIP-Seq data for detecting the AR-binding sites in LNCaP cells was accessed from GSE55007. BedGraph files obtained were visualized by Integrative Genomic Browser. ChIP assay was performed following our established protocol^46^. Briefly, cultured cells were cross-linked with 1% formaldehyde and then stopped with 125 mM glycine. The cells were washed twice and lysed with lysis buffer. Nuclei pellets were collected after centrifugation and then resuspended and incubated in the nuclear lysis buffer. Chromatin was sonicated using a microtip soniactor (Model 120, Fisher) to obtain 200–500 bp DNA fragments. The chromatin was then centrifuged at 4 °C to remove the debris and 5% input was collected. Supernatants were incubated with agarose/protein A or G beads (Thermo Fisher) and centrifuged to eliminate nonspecific binding. The cleared immunoprecipitated chromatin complexes were incubated with 2 μg of either primary or isotype control antibodies overnight at 4 °C, and 20 μl of magnetic agarose/protein A or G beads were added for a 3-h incubation. Beads were washed with washing buffer five times. The immunocomplex was eluted using the elution buffer. DNA was isolated using phenol–chloroform–isoamylol alcohol extraction. Precipitated DNA was washed with 70% ethanol, air-dried, and resuspended in nuclease-free water (Ambion). Antibodies and ChIP-PCR primers are listed in the Supplementary file.

### Dual-luciferase reporter assay

The pGL3-promoter plasmid (Promega) was employed as a backbone for luciferase reporter construction. Amplified and purified PCR products (ARE1, ARE2, and ARE1 with point mutations or deletion) were, respectively, inserted into the multiple cloning sites of the backbone plasmid for the establishment of pGL3-ARE1, pGL3-ARE2, pGL3-ARE1mut, and pGL3-ARE1del constructs following our previous report^35^. For dual-luciferase reporter assay, briefly, LNCaP cells were plated at 30–40% confluency in a 24-well plates, and were transfected with 250 ng of luciferase reporter and 5 ng of pGL4.70 Renilla reporter using Lipofectamine 3000. Cells were treated with either EtOH or 10 nM R1881 (Sigma-Aldrich) overnight. Cells were harvested using the lysis buffer of Dual-Glo Luciferase assay system (Promega). Firefly and Renilla luciferase activities were tested according to the manufacturer’s instructions using the Infinite 200Pro Microplate Reader (Tecan). For each sample, transfection efficiency was normalized to Renilla luciferase activity.

### Mouse tumor model studies

All animal procedures were conducted in accordance with the NIH Guidelines of Care and Use of Laboratory Animals and approved by the Animal Ethics Committee of The Third Xiangya Hospital of CSU (2020sydw0041). Briefly, 1 × 10^6^ PCa cells were resuspended in 0.1 ml of saline with 50% Matrigel (Corning) and randomly injected subcutaneously into both sides of flank regions of 5-week-old male athymic nude mice (nonobese diabetic/severe combined immunodeficiency). For evaluating ENZ (GLPBIO) resistance, when the tumors were palpable, mice bearing C4-2 cells (control/EHF knockdown) were treated with ENZ (30 mg/kg, intraperitoneal injection, every other day). Tumor volume was measured every third day with calipers and calculated by the formula: length × width^2^/2. At the endpoint, all tumors were harvested and disposed of for immunoblotting and IHC staining.

### IHC staining

IHC study was performed as previously described^46^. Briefly, the tumors were fixed in 4% paraformaldehyde solution and embedded in paraffin. The primary antibodies were used at the optimal dilution. The biotinylated secondary antibodies were diluted properly for recognizing primary antibodies. The slides were then colored with DAB, followed by hematoxylin counterstaining, and finally observed under a microscope. Information on antibodies is listed in the Supplementary file.

### Statistics

All data represent three or more times of experiments. The data values were presented as the mean ± SD. All statistical analyses were performed using the GraphPad Prism 5.01 software (GraphPad Software, CA, USA). Differences in mean values between two groups were analyzed by two-tailed Student’s *t* test or one-way analysis of variance, followed by Dunnett’s *t* test. Correlation between two expression groups was determined by Pearson’s *r* correlation coefficient, and overall survival was measured by Kaplan–Meier. *P* < 0.05 was taken as a statistical significance.

## Supplementary information

Supplementary files

Supplementary tables
