# Supplementary material for: Loss of EHF facilitates the development of treatment-induced neuroendocrine prostate cancer
Source: Cell Death Dis. 2021 Jan 5;12(1):46. doi: 10.1038/s41419-020-03326-8 (PMC7790822; doi:10.1038/s41419-020-03326-8)
Supplement: Supplementary file 1 — Supplementary files [file 41419_2020_3326_MOESM1_ESM.pdf]

Loss of EHF promotes the development of treatment-induced neuroendocrine prostate cancer

Zhi Long[1], Liang Deng[1], Chao Li[1], Qiangrong He[1], Yao He[2], Xiheng Hu[2], Yi Cai[2], Yu Gan[2]

1. Andrology Center, Department of Urology, the Third Xiangya Hospital, Central South University, Changsha, Hunan, P.R. China, 410013;
2. Department of Urology, Xiangya Hospital, Central South University, Changsha, Hunan, P.R. China, 410008;

Corresponding author: Yu Gan, M.D.
87 Xiang Road, Changsha
Hunan, P.R. China, 410008
Tel: +86 15111140206
Email: 148302039@csu.edu.cn

Disclosure Statement: The authors have declared that no conflict of interest exists.

Supplementary Materials

I. Antibody Information

II. RNA and Plasmid Information

III. Primers for qPCR

IV. Supplementary Figures

V. References

I. Antibody Information

Antibody	Vendor	Catalogue Number	Application	Dilution
AR	Proteintech	22089-1-AP	WB	1:1000
EHF/ESE-3	Proteintech	67125-1-Ig	WB	1:1000
	Abcam	Ab272671	IHC	1:200
ENO2	Proteintech	10149-1-AP	WB	1:1000
			IHC	1:200
CHGA	Proteintech	60135-1-Ig	WB	1:2000
H3K27me3	Omnimabs	OM256819	WB	1:1000
Histone H3	CST	4499	WB	1:1000
EZH2	Proteintech	21800-1-AP	WB	1:1000
SOX2	Abcam	Ab97959	WB	1:1000
			IHC	1:200
Vinculin	Sigma Aldrich	V9131-2ML	WB	1:2000
Ki-67	Genetex	GTX16667	IHC	1:100
AR	CST	5153	ChIP	1:100
H3K9Ac	CST	9649	ChIP	1:50
Rabbit IgG	Invitrogen	10500C	ChIP	1:50/100

Ⅱ. RNA and Plasmid Information

Reagent	Provider	Catalogue #
ON-TARGETplus Human AR siRNA	Dharmacon	L-003400-00-0005
ON-TARGETplus Human EZH2 siRNA	Dharmacon	L-004218-00-0005
ON-TARGETplus Non-targeting Pool	Dharmacon	D-001810-10-05
TRC Lentiviral Human EHF shRNA	Dharmacon	RHS4533-EG26298
pGL3-Promoter Vector	Promega	E1761
pCMV-hAR	Addgene; pCMV-hAR was a gift from Elizabeth Wilson	89078

Ⅲ. Primers for qPRC

Primer name	Forward Primer Sequence (5’-3’)	Reverse Primer Sequence (5’-3’)
CHGA	TAAAGGGGATACCGAGGTGATG	TCGGAGTGTCTCAAAACATTCC
ENO2	CTGTGGTGGAGCAAGAGAAA	ACACCCAGGATGGCATTG
PSA	AGTGCGAGAAGCATTCCCAAC	CCAGCAAGATCACGCTTTTGTT
SLIT2	CGGAGCAGCAAGCTAAAGAA	GCGACAGGGACAGCATCT

DAB2IP	CTGAGCGGGATAAGTGGATGG	AAACATTGTCCGTCTTGAGCTT
ADRB2	TTCTTGCTGGCACCCAATA	GCCAGGACGATGAGAGACAT
GAPDH	GGACCTGACCTGCCGTCTAGAA	GGTGTCGCTGTTGAAGTCAGAG
ARE1 (ChIP-Seq)	GAAGCCTGGCATGTTGTACTTG	GGTAGGAACAATTGGAGGGAAGT
ARE2 (ChIP-Seq)	TGGAGAACTGGCCCATATTGTT	TGAACCAGTGCCAAATCTGTCT
PSA (ChIP-Seq)	CCTAGATGAAGTCTCCATGAGCTACA	GGGAGGGAGAGCTAGCACTTG

IV. Supplementary Figures

Supplementary Figure 1

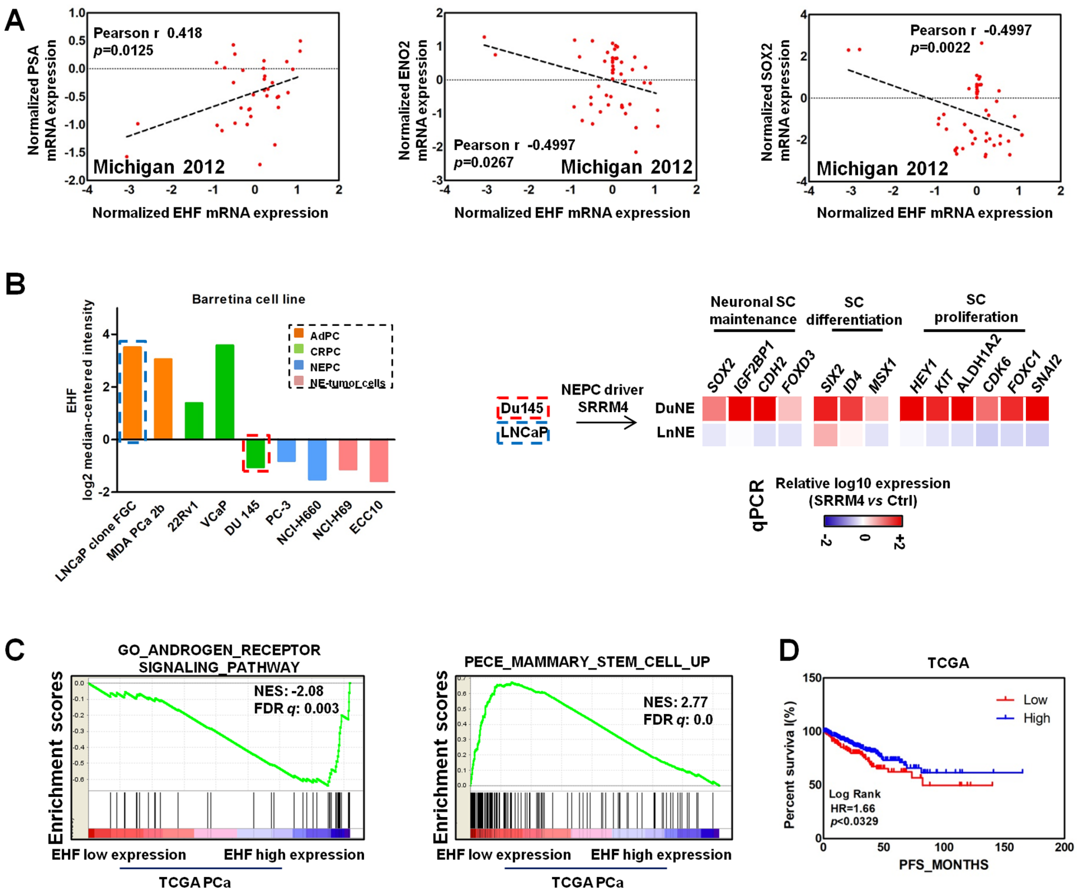

Figure. S1. (A) Pearson correlation between EHF and PSA, EHF and ENO2, and EHF and SOX2 expressions was determined using transcriptomic data of Michigan 2012 cohort[1]. **(B)** EHF expression in different cancer cell lines from Barretina *et al.* 2016[2]. Results are presented as mean ± SD (left). The mRNA expression of the genes with functions on neuronal stem cell maintenance, stem cell differentiation and stem cell proliferation in the SRRM4-overexpressing DU145 and LNCaP cells was compared to that of their respective control cells via qPCR. Heat maps represent the relative fold change in log10. **(C)** GSEA analysis indicated that PCa tumors with low EHF expression in TCGA clinical prostate cancer cohort[3] had similar characteristics with t-NEPC in alleviating the dependency on AR signaling and gaining stem-cell features. **(D)** Kaplan-Meier analysis indicated that PCa tumors with low EHF

expression had a poor overall survival. Note: AdPC, prostate adenocarcinoma; CRPC, castration-resistant prostate cancer; NEPC, neuroendocrine prostate cancer; NE, neuroendocrine; SC, stem cell; PCa, prostate cancer; NES, Normalized enrichment score; FDR, false discovery rate; PFS, progression-free survival; HR, hazard rate.

Supplementary Figure 2

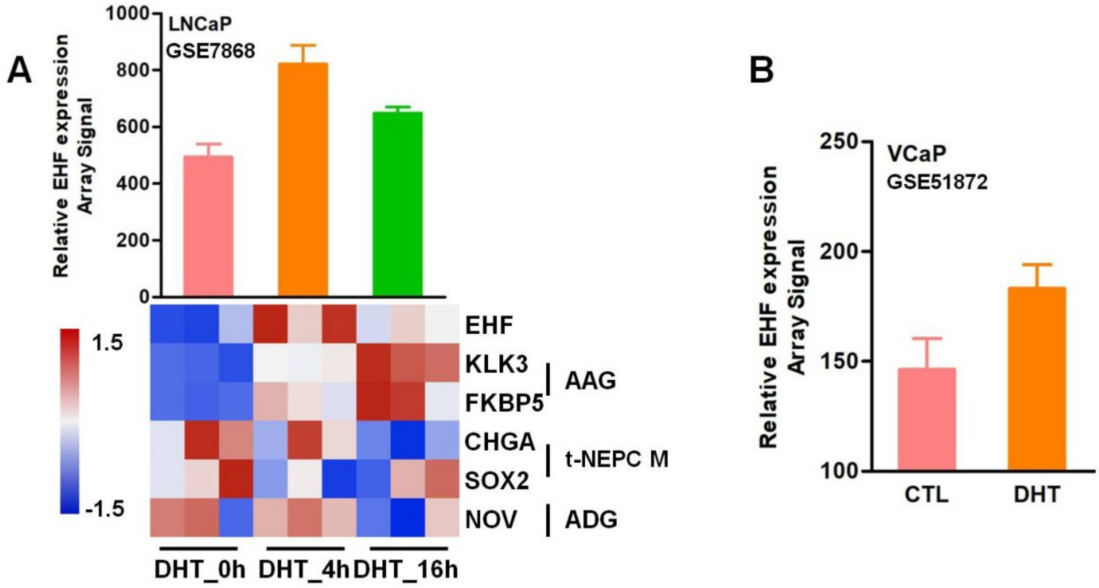

Figure. S2. (A) Bar graph showing EHF expression (Top) and heatmap of AR-associated genes including EHF, t-NEPC markers and an androgen-depressed gene in DHT treated LNCaP cells (GSE7868). **(B)** Bar graph showing EHF expression in DHT or ENZ treat VCaP cells (GSE51872). Bar graphs show means ± SD. Note: AAG, AR-associated genes; M, markers; ADG, androgen-depressed gene; AD, androgen deprivation; CTL, control; DHT, double hydrogen testosterone.

Supplementary Figure 3

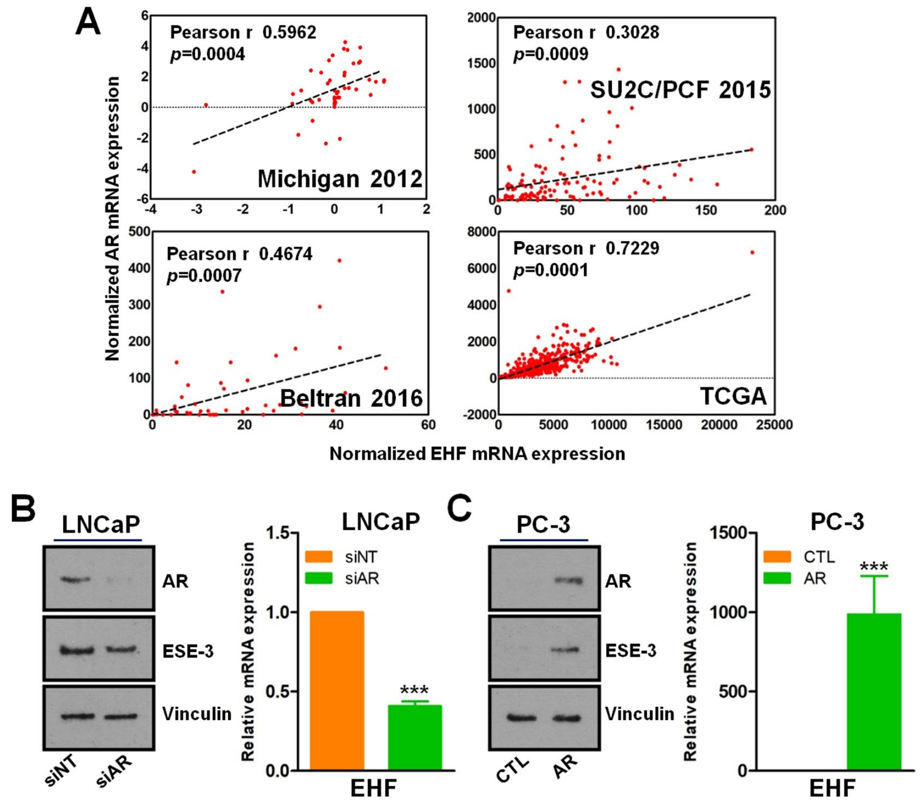

Figure. S3. (A) EHF and AR are positive correlated in Michigan 2012[1],SU2C/PCF 2015[4], Beltran 2016[5] and TGCA[3] clinical protate cancer cohort. **(B)** AR and EHF levels in LNCaP cells with/without AR knockdown were measured by qPCR and immunoblotting. **(C)** AR and EHF levels in PC-3 cells with/without AR overexpression were measured by qPCR and immunoblotting. The two-tailed Student's t-test was used to compare results between two groups with *** denoting *p*< 0.001. Bar graphs show means ± SD. Note: TCGA, the cancer genome atlas; CTL, control; NT, non-target.

Supplementary Figure 4

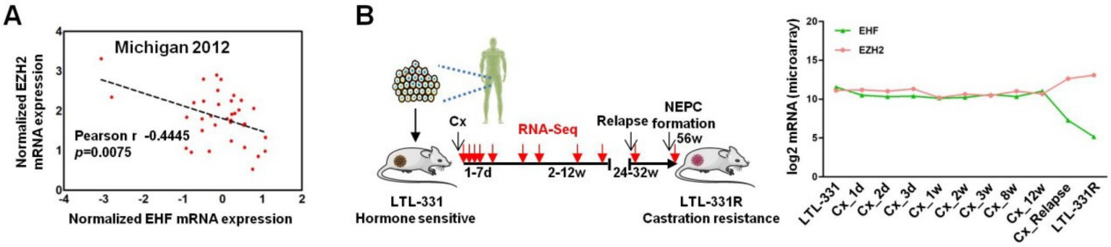

Figure. S4. (A) Pearson correlation between EHF and EZH2 was determined using transcriptomic data of Michigan 2012 cohort[1]. **(B)** EHF and EZH2 mRNA expressions during progression of AdPC (LTL-331) to t-NEPC (LTL-331R) by castration surgery to the host mice[6] were plotted. Note: Cx, castration.

Supplementary Figure 5

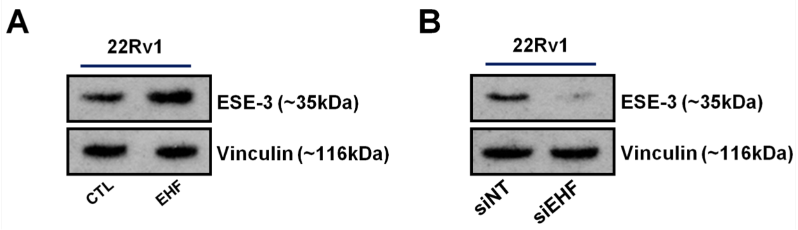

Figure. S5. (A) ESE-3 expression level in 22Rv1 cells with/without EHF overexpression was measured by immunoblotting. **(B)** ESE-3 expression level in 22Rv1 cells with/without EHF knockdown was measured by immunoblotting.

Supplementary Figure 6

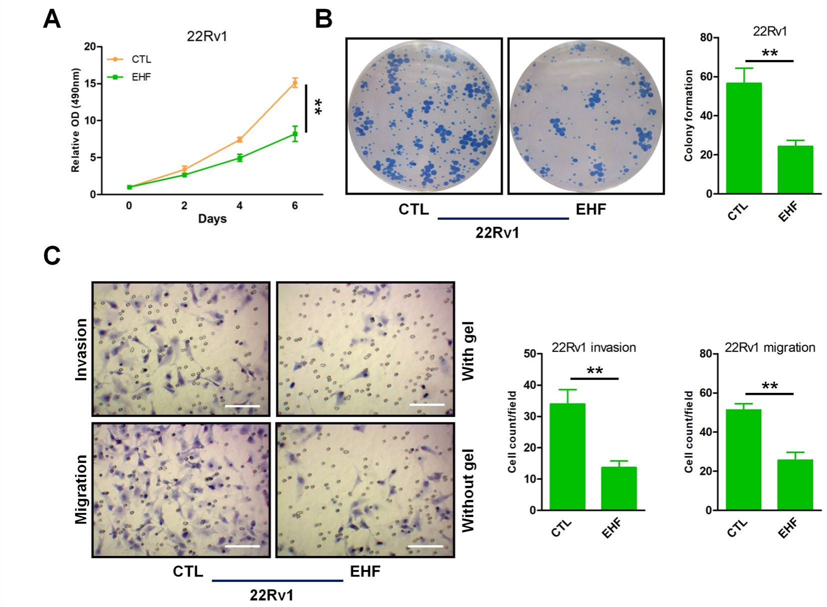

Figure. S6. (A) MTS assays measured cell proliferation of 22Rv1 cells with/without EHF

overexpression. **(B)** Same cells as in **A** were used to perform colony formation assays. Representative images are shown. **(C)** Same cells as in **A** were used to perform cell invasion and migration assays in transwell chambers with/without matrigel coated. Representative images are shown. Scale bar 100μm. Two-tailed Student's t-test was used to compare results between two groups with ** denoting $p< 0.01$. Experiments were performed with three biologically independent samples. Bar graphs show means ± SD. Note: OD, optical density.

Supplementary Figure 7

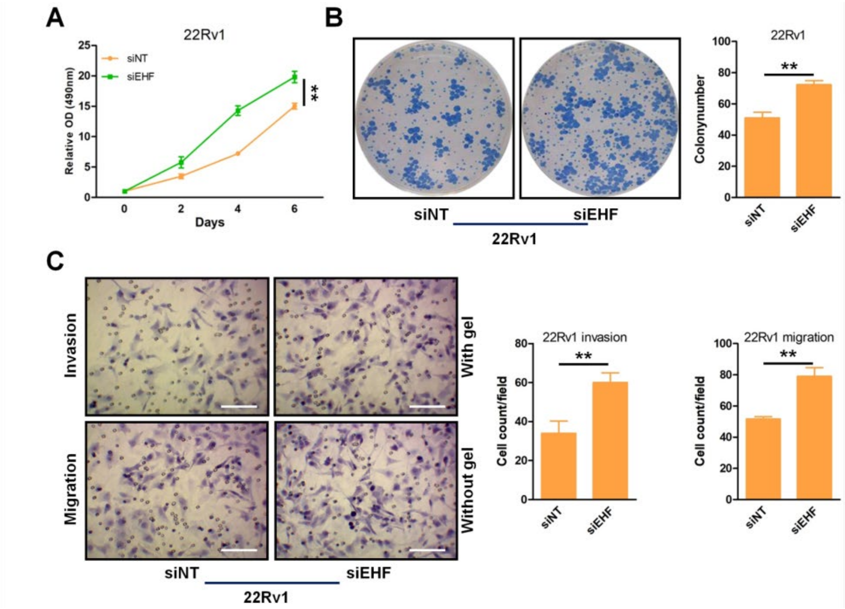

Figure. S7. (A) MTS assays measured cell proliferation of 22Rv1 cells with/without EHF knockdown. **(B)** Same cells as in **A** were used to perform colony formation assays. Representative images are shown. **(C)** Same cells as in **A** were used to perform cell invasion and migration assays in transwell chambers with/without matrigel coated. Representative images are shown. Scale bar 100μm. Two-tailed Student's t-test was used to compare results between two groups with ** denoting $p< 0.01$. Experiments were performed with three biologically independent samples. Bar graphs show means ± SD. Note: OD, optical density.

V. References

1. Grasso CS*, et al.* The mutational landscape of lethal castration-resistant prostate cancer. *Nature* **487**, 239-243 (2012).
2. Barretina J*, et al.* The Cancer Cell Line Encyclopedia enables predictive modelling of anticancer drug sensitivity. *Nature* **483**, 603-607 (2012).
3. Cancer Genome Atlas Research N. The Molecular Taxonomy of Primary Prostate Cancer. *Cell* **163**, 1011-1025 (2015).
4. Robinson D*, et al.* Integrative Clinical Genomics of Advanced Prostate Cancer. *Cell* **162**, 454 (2015).
5. Beltran H*, et al.* Divergent clonal evolution of castration-resistant neuroendocrine prostate cancer. *Nature medicine* **22**, 298-305 (2016).
6. Lin D*, et al.* High fidelity patient-derived xenografts for accelerating prostate cancer discovery and drug development. *Cancer research* **74**, 1272-1283 (2014).
